# Improvement of Human Keratinocyte Migration by a Redox Active Bioelectric Dressing

**DOI:** 10.1371/journal.pone.0089239

**Published:** 2014-03-03

**Authors:** Jaideep Banerjee, Piya Das Ghatak, Sashwati Roy, Savita Khanna, Emily K. Sequin, Karen Bellman, Bryan C. Dickinson, Prerna Suri, Vish V. Subramaniam, Christopher J. Chang, Chandan K. Sen

**Affiliations:** 1 Department of Surgery, Davis Heart and Lung Research Institute, The Ohio State University Wexner Medical Center, Columbus, Ohio, United States of America; 2 Department of Mechanical & Aerospace Engineering, The Ohio State University, Columbus, Ohio, United States of America; 3 Department of Chemistry and Molecular and Cell Biology, Howard Hughes Medical Institute, University of California, Berkeley, California, United States of America; National Institutes of Health, United States of America

## Abstract

Exogenous application of an electric field can direct cell migration and improve wound healing; however clinical application of the therapy remains elusive due to lack of a suitable device and hence, limitations in understanding the molecular mechanisms. Here we report on a novel FDA approved redox-active Ag/Zn bioelectric dressing (BED) which generates electric fields. To develop a mechanistic understanding of how the BED may potentially influence wound re-epithelialization, we direct emphasis on understanding the influence of BED on human keratinocyte cell migration. Mapping of the electrical field generated by BED led to the observation that BED increases keratinocyte migration by three mechanisms: (i) generating hydrogen peroxide, known to be a potent driver of redox signaling, (ii) phosphorylation of redox-sensitive IGF1R directly implicated in cell migration, and (iii) reduction of protein thiols and increase in integrin_αv_ expression, both of which are known to be drivers of cell migration. BED also increased keratinocyte mitochondrial membrane potential consistent with its ability to fuel an energy demanding migration process. Electric fields generated by a Ag/Zn BED can cross-talk with keratinocytes via redox-dependent processes improving keratinocyte migration, a critical event in wound re-epithelialization.

## Introduction

The presence of electric fields and natural direct currents, in the vicinity of regenerating limbs, driven by so-called ‘skin batteries’, is essential for normal regeneration, and their reversal induces degeneration [1], [2]. These currents originate from the epidermis and when artificially introduced into species such as a frog, which are not natural regenerators, partial regeneration is initiated [1], [3], [4]. During wounding, the trans-epithelial potential (TEP) collapses to zero at the wound site and the TEP in the surrounding intact epithelium generates an endogenous electric field around and directed into the site of injury [3]. Cells within the wound electric field respond with a variety of biological and functional responses [5]. An externally applied electric field can then result in an electric current being driven along the wound surface, enhancing the endogenous electric field and arguably augmenting the healing processes [5], [6], [7], [8], [9], [10], [11], [12]. Electric fields have been reported to direct cell migration in many different cell types [11], [13], [14], activate signaling pathways such as cdc42p, Rho/Rac, PI3K/PTEN and phosphatidylinositol (PIP) [11], [15], [16], [17], activation of epithelial sodium channels [18], cellular electrotaxis of macrophages [19], [20], neutrophils [19], [21] and fibroblasts [22], [23], [24], [25], increase production of ATP and DNA [19], [26], [27], [28], increase collagen secretion by fibroblasts [22], [29] and increase blood flow and capillary density [30], [31], [32]. Although there are some outcome-based and mechanistic evidence supporting electrical stimulation (ES) promoting wound healing [11], a better understanding is lacking because of limitation in standardized procedure of application of ES to wounds. Some of the devices currently in use in the clinic for wound healing are Provant (Regenesis Biomedical), Ivivi SofPulse (Ivivi Health Sciences) and Posifect (Biofisica). In this work, we provide the first evidence characterizing a novel FDA approved silver-zinc coupled bioelectric dressing (BED) which is currently being used in clinical wound care. The advantage of this device is that it is wireless and has no need for an external power source, can be cut to the size of the wound and conforms to irregular surfaces and provides an electrical field in the range of the physiologic fields [33], [34] found at healing wound edges. To develop a mechanistic understanding of how the BED may potentially influence wound re-epithelialization, a key event in wound healing, we directed emphasis on understanding the influence of BED on human keratinocyte cell migration.

## Materials and Methods

### Scanning Electron Microscopy (SEM) and Energy Dispersive X-ray Spectroscopic (EDS) Analysis

SEM imaging was performed on the silver and zinc dots (Quanta 200, FEI Inc., Hillsboro, OR). The chemical composition was studied using Energy Dispersive X-ray (EDX or EDS) spectroscopy (Edax Inc., 91 McKee Drive, Mahwah NJ). The EDS equipment used a 10 square millimeter Sapphire x-ray detector with a super ultra thin window (SUTW), digital pulse processor and Genesis software.

### Cell culture

Immortalized HaCaT human keratinocytes (provided kindly by Dr. NE Fusenig of German Cancer Research Center, Heidelberg) [35] were grown in Dulbecco's low-glucose modified Eagle's medium (Life Technologies, Gaithersburg, MD, U.S.A.) supplemented with 10% fetal bovine serum, 100 U/ml penicillin, and 100 µg/ml streptomycin. The cells were maintained in a standard culture incubator with humidified air containing 5% CO2 at 37°C as described previously [36].

### Ag/Zn Bioelectric dressing

A bioelectric dressing, Procellera designed and provided by Vomaris Innovations, Inc. was used. A polyester cloth printed with polyvinylchloride with the same design was used as a control. For all assays, cells were seeded in a 35 mm plate with 7 ml media and the dressing was cut into a circle of diameter of 25 mm and kept floated on the surface of the media.

### Scratch assay

A cell migration assay was performed using culture inserts (IBIDI, Verona, WI) according to the manufacturer's instructions. Briefly, the cells were seeded in the chambers in such a way that a confluent monolayer is formed in the presence of the insert. Removal of the insert generated a gap in the monolayer. Migration of cells across that gap was studied using time-lapse microscopy. As required, cells were grown in the presence of BED or a sham control fabric. Cell migration was measured using time-lapse phase-contrast microscopy following withdrawal of the insert. Images were analyzed using the AxioVision Rel 4.8 software. *N-Acetyl Cysteine Treatment*. Cells were pretreated with 5 mM of the thiol antioxidant N-acetylcysteine (Sigma) for 1 h before start of the scratch assay. *IGF-1R inhibition*. When applicable, cells were preincubated with 50 nM IGF-1 R inhibitor, picropodophyllin (Calbiochem, MA) [37] just before onset of the scratch Assay.

### Direct H_2_O_2_ measurements

H_2_O_2_ levels in wound fluid were measured using a real-time electrochemical H_2_O_2_ measurement. The Apollo 4000 system (WPI, Sarasota, FL, USA) was used for analysis. H_2_O_2_ was measured using the ISO-HPO-2 2.0-mm stainless steel sensor, with replaceable membrane sleeves and an internal refillable electrolyte. This electrode technology includes a H_2_O_2_-sensing element and separate reference electrode encased within a single Faraday-shielded probe design (WPI) [38].

### Cellular H_2_O_2_ Analysis

To determine intracellular H_2_O_2_ levels, HaCaT cells were incubated with 5 µM PF6-AM in PBS for 20 min at room temperature (PF6-AM is a fluorescent probe provided kindly by Prof. Christopher J. Chang and Bryan C. Dickinson at University of California, Berkeley). After loading, cells were washed twice to remove excess dye and visualized using a Zeiss Axiovert 200M microscope.

### Catalase gene delivery

AdCatalase viral vectors were provided by Dr. John F. Engelhardt of the University of Iowa. HaCaT cells were transfected with 2.3×10^7^ pfu AdCatalase or with the empty vector as control in 750 µL of media. Subsequently, 750 µL of additional media was added 4 h later and the cells were incubated for 72 h.

### RTK Phosphorylation Assay

Human Phospho-Receptor Tyrosine Kinase phosphorylation was measured using Phospho-RTK Array kit (R & D Systems).

### ELISA

Phosphorylated and total IGF-IR were measured using a DuoSet IC ELISA kit from R&D Systems.

### Determination of Mitochondrial Membrane Potential

Mitochondrial membrane potential ΔΨ was measured in HaCaT cells exposed to the BED or placebo using TMRM/PMPI or JC-1 (MitoProbe JC-1 Assay Kit for Flow Cytometry, Life technologies), per manufacturer's instructions for flow cytometry as reported previously [39], [40], [41], [42].

### Immunocytochemistry

HaCaT Cells were fixed with 4% paraformaldehyde, permeabilized with 0.1% Triton-X 100, blocked with normal goat serum and incubated with primary antibody. Integrin αV was visualized with the help of Alexa Fluor 568 dye-conjugated antibody against mouse, and counterstained with DAPI. Images were captured using a Zeiss Axiovert 200M microscope supported by Axiovision Rel 4.8 software. *Integrin αV Expression*. HaCaT cells were grown under the BED or placebo and harvested 6 h after removing the IBIDI insert. Staining was done using antibody against integrin αV (Abcam, Cambridge, MA).

### Flow Cytometric Determination of Thiols Using Bimane Probe

Bimane-loaded cells were excited using a 20 mW powered UV line of an argon ion laser set at 350 nm in a BDFACSAria flow cytometer. Fluorescent emission from cellular sulfhydryl reacted bimane was recorded using a 450 nm bandpass filter. A morphometrically homogenous cell population, typically representing ≈90% of the total population, was gated. Data were collected from at least 10,000 cells at a flow rate 250–300 cells/s. When regular-sized and shrunken cell populations were studied, two gates containing a representative population of each cell type were constructed, and bimane emission from the two gated cell population were collected simultaneously [43].

## Results and Discussions

### Characterization of the Ag/Zn BED

**Figure 1:**
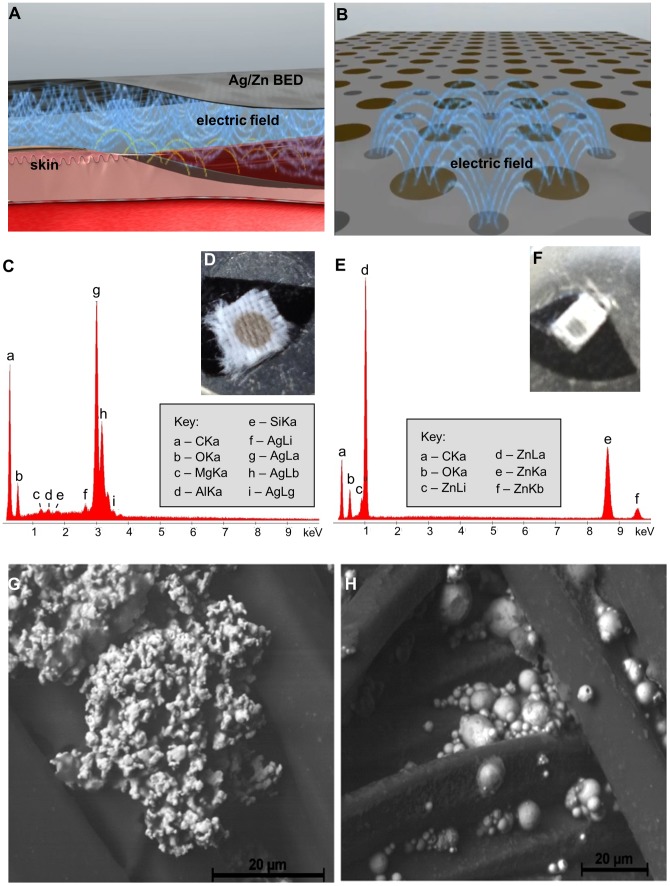
Characterization of the Ag/Zn BED. (**A, B**) Schematic diagram of the design, application and electric fields generated by the bioelectric dressing, (**C**) EDS spectrum of a silver dot on a BED (**D**) EDS spectrum of a zinc dot on a BED. (**E**) Photographs of silver and (**F**) zinc dots of the BED mounted using carbon tape in an aluminum holder for energy dispersive spectroscopic (EDS) X-ray analysis. (**G**) Scanning electron microscope image of a silver dot on a BED (**H**) Scanning electron microscope image of a zinc dot on a BED.

The Ag/Zn BED is made of polyester printed with dissimilar elemental metals. It comprises alternating circular regions of silver and zinc dots, along with a proprietary, biocompatible binder added to lock the electrodes to the surface of a flexible substrate in a pattern of discrete reservoirs. The metals placed in proximity of about 1 mm to each other thus forming a redox couple and generating an ideal potential on the order of 1 Volt. (**Figure 1A,B**). The Ag dot is 2 mm and the Zn dot is 1 mm in diameter, respectively. However, it must be pointed out that these metallic regions are not contiguous. They are reservoirs of 2 to 10 micron elemental grains (as evident from the SEM) embedded onto the polyester fibers in that location. **Figures 1C–H** show photographs, scanning electron microscope (SEM) images, and energy dispersive spectroscopic (EDS) X-ray analysis of small regions within the individual silver and zinc dots of the BED. It can be seen from these results that the large dot contains oxides of silver while the small dot contains oxides of zinc.

Elemental Ag and Zn used to make the dots in the BED are 99.999% pure as acquired from commercial sources. The EDS spectra reveal their presence as well as their oxides and other elements such as carbon, aluminum, silicon, and magnesium (**Figure 1**). The carbon peak arises from the carbon black tape used to mount the sample onto the holder in the SEM. The much smaller peaks in the EDS spectrum corresponding to Mg, Al, and Si arise from the elements in the substrate holder underneath the sample. Mg, Si, and Al (as well as C, Na, Cl, and Ca) can also arise from dust particles (which consist of silicates and carbonates among other compounds) which appear from handling the sample before placing it in the SEM.

When the metal electrodes are in contact with a conducting medium, the electric field present between positive and negative electrodes, leads to ambipolar diffusion [44]. It refers to a collective diffusion where electrons and positive ions collectively diffuse with a diffusivity smaller than that of electrons but larger than that of positive ions. This can happen between positive and negative ions as well of different masses. While the cited reference [44] is for plasmas, the physics holds for liquids and solids as well [45].

We hypothesize that when the BED is in contact with an aqueous solution, the silver is the positive electrode (cathode) and is reduced while zinc is the negative electrode (anode) and is oxidized. When these electrodes contact a conducting fluid, such as wound exudate, an exogenous hydrogel, saline, or even pure water, a set of redox reactions is expected to occur. Beginning with the silver oxide electrode, OH^−^ ions are generated from:
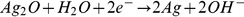
(1)

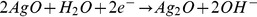
(2)


The OH^−^ ions then migrate to the zinc dots and are consumed by:
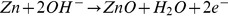
(3)As is evident in the scientific literature on the electrochemistry of silver zinc batteries [46], both Ag2O and AgO react with H_2_O via these reactions. These reactions however should not be confused with solubility of Ag, as they are electrochemical reactions that happen at the oxidized surface of silver. Once the oxide is consumed, the reactions stop, unless the oxide is regenerated by other means. No applied electric field is necessary to drive these reactions.

In addition to OH^−^ ions, there may be other ions present in the exudate, such as H^+^, Na^+^, Ca^++^, Cl^−^, Mg^++^, and K^+^, to name only a few. Moreover, these ions are hydrated but may still react and recombine to form other chemical species. Consequently, migration of a particular ionic species from one electrode to the other may be impeded by attachment losses (such as on charged domains of protein surfaces), recombination, and chemical reaction, rendering the determination of any current flow complicated.

### Magnitude and extent of electric fields adjacent to Ag/Zn BED

**Figure 2:**
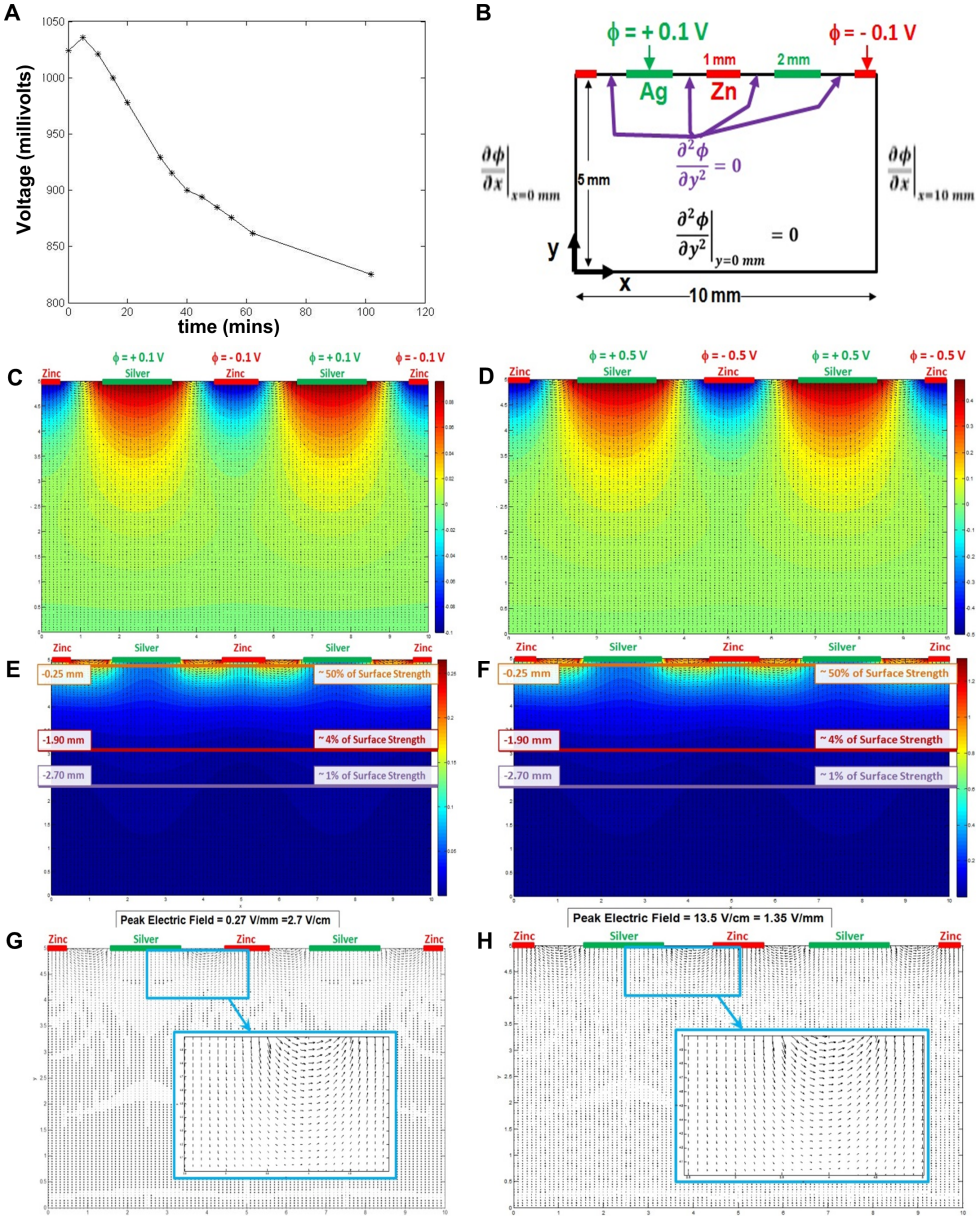
Experimental determination of electric fields generated by the bioelectric dressing. (**A**) Voltage measurements from an experiment with a Zn (99.994% pure) and silver (99.998% pure) foil immersed in 100 mL of de-ionized water. (**B**) Schematic showing the computational grid and boundary conditions used to calculate the magnitude and extent of the electric fields under the bioelectric dressing presumed to be in contact with H_2_O. (**C, D**) Contour plots of calculated values of the electric potential for 0.2 V and 1 V potential difference, respectively. (**E, F**) Electric fields from the solution of the 2-D Laplace equation for the particular case of 0.2 V and 1 V potential difference, respectively between the silver and the zinc dots. (**G,H**) Expanded view of the electric field vectors between adjacent pairs of electrodes for potential difference of 0.2 V and 1 V respectively.

Simple measurement of the potential difference was performed between adjacent zinc (99.994% pure) and silver (99.998% pure) foil immersed in 100 mL of de-ionized water was performed using an oscilloscope, across a 10 megaOhm load (Oscilloscope) (**Figure 2A**). However, non-intrusive measurement of the electric field arising from contact between the BED and liquid medium is difficult. Therefore, the magnitude of the resulting electric field and its extent into the liquid medium or wound must be determined using numerical calculations.

We consider a line of alternating silver and zinc dots on the bioelectric dressing in contact with H_2_O. The silver dots are assumed to be 2 mm in diameter, the zinc dots 1 mm in diameter, and the separation between the dots is 1 mm. The magnitude and extent of the electric field in the vicinity of a pair of zinc and silver dots can be determined by solving the two-dimensional Laplace equation for the potential, assuming no net charge density:
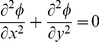
(4)where *ϕ* is the electric potential measured in Volts and the coordinate system is shown in **Figure 2B**. The electric field is given by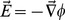. Two distinct cases with potential difference of 0.2 V or 1 V is assumed between silver and zinc dots. By symmetry, the x-component of the electric field E_x_ = 0 at the lateral boundaries (x = 0 and x = 10 mm), and implicit extrapolation (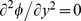) is applied at all other boundaries where the potential is presumed unknown. Equation (4) is solved using finite differences, and the resulting system of coupled linear equations is solved using the Gauss-Seidel iterative method. A grid-independent solution is obtained for a 101×101 grid used to discretize the 10 mm×5 mm domain. The results of the numerical calculation are shown in **Figures 2C–H**. **Figures 2C,D** display contour plots of the electric potential and **Figures 2E,F** illustrate contour plots of the electric field magnitudes. **Figures 2G,H** show an expanded view of the electric field lines between adjacent electrode pairs. The maximum values of the electric field in the two cases (2.7 V/cm for Δϕ = 0.2 V and 13.5 V/cm for Δϕ = 1 V) occur at the surface of the BED, between the silver and zinc dots. For the particular case of a 0.2 V potential difference between adjacent silver and zinc dots, it is observed that the strength of the electric potential is diminished by 50% within 0.55 mm from the surface of the bioelectric dressing while the magnitude of the electric field is diminished by 50% within 0.25 mm from the surface of the bioelectric dressing. For Δϕ = 1 V, these calculations indicate that while the presence of the electric potential (and electric field) extends further away from the bioelectric dressing, the strength of the electric field is still reduced by 50% within 0.25 mm as in the case of a potential difference of 0.2 V.

### Ag/Zn BED accelerates migration of human keratinocytes

**Figure 3:**
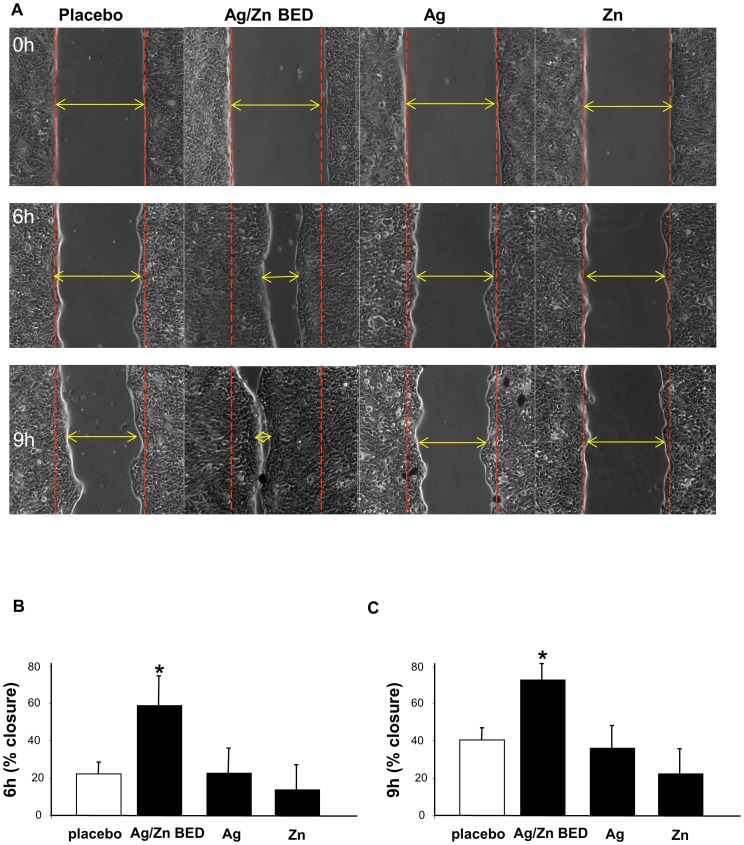
Increased rate of migration of human keratinocytes under an Ag/Zn bioelectric dressing (BED). (**A**) HaCaT cells were treated with Ag/Zn BED or polyester printer printed with only silver or only zinc for 24 h followed by scratch assay and migration of cells was observed at 6 h and 9 h following scratch. (**B**) % closure at 6 h, (**C**) % closure at 9 h. BED significantly increased rate of migration while placebo or silver alone or zinc alone did not change cell migration. (n = 4).

One key aspect of wound closure is re-epithelialization, in which keratinocyte migration is a critical event. Exposure to Ag/Zn BED significantly accelerated keratinocyte cell migration (**Figure 3A–C**). Replacing the Ag/Zn redox couple with Ag or Zn alone failed to reproduce the effect on keratinocyte migration. These observations directly implicate the redox couple property of BED in facilitating cell migration.

The patterned design of the BED creates a complex pattern of the electrical field at the device surface which enables cells to migrate in the gap between the electrodes. Thus, the BED generates multiple regions of potential difference over the entire surface of the wound, enabling an electric field to be applied across the wound without the need for external power. The calculated values of the electric field from the BED are consistent with the magnitudes that are typically applied in classical electrotaxis experiments, suggesting that cell migration observed with the bioelectric dressing is likely contributed by electrotaxis. The magnitudes of the voltage and current will all scale with the area of the electrodes in contact with the water, so the size of the dots and the distances between them in the Ag/Zn BED are important parameters to optimize for wound healing. However, the extent of penetration of the electric field will not change. These calculations serve to determine the extent of the electric field arising from the bioelectric dressing and can aid in optimizing the size of the silver and zinc dots as well as their separation to deliver a maximum effect on cell migration.

The direction of the electric field vector is by convention, from positive to negative. The silver dot is the positive electrode and the zinc dot is the negative electrode. The field lines therefore begin at the silver electrode and end at the zinc electrode. Prior works [33] show that keratinocytes migrate toward the negative electrode (in our case, zinc). The migration in the present experiments, therefore, should be towards the negative, zinc pole. However, we have not reported observations related to the direction of cell motility in this paper. The placement of the the dots (positive and negative) is more complex than a simple parallel array where a straight vector from positive to negative electrode may be identified. There is consequently no uni-directional gradient with respect to the axis of the gap or simulated wound in the assay. The patterned approach presents a complex electric field distribution that can move with motion of the dressing. In addition, cells are present both at the wound edge as well as in the wound itself; therefore galvanotactic directionality may be localized at a cellular level.

### Ag/Zn BED generates H_2_O_2_

**Figure 4:**
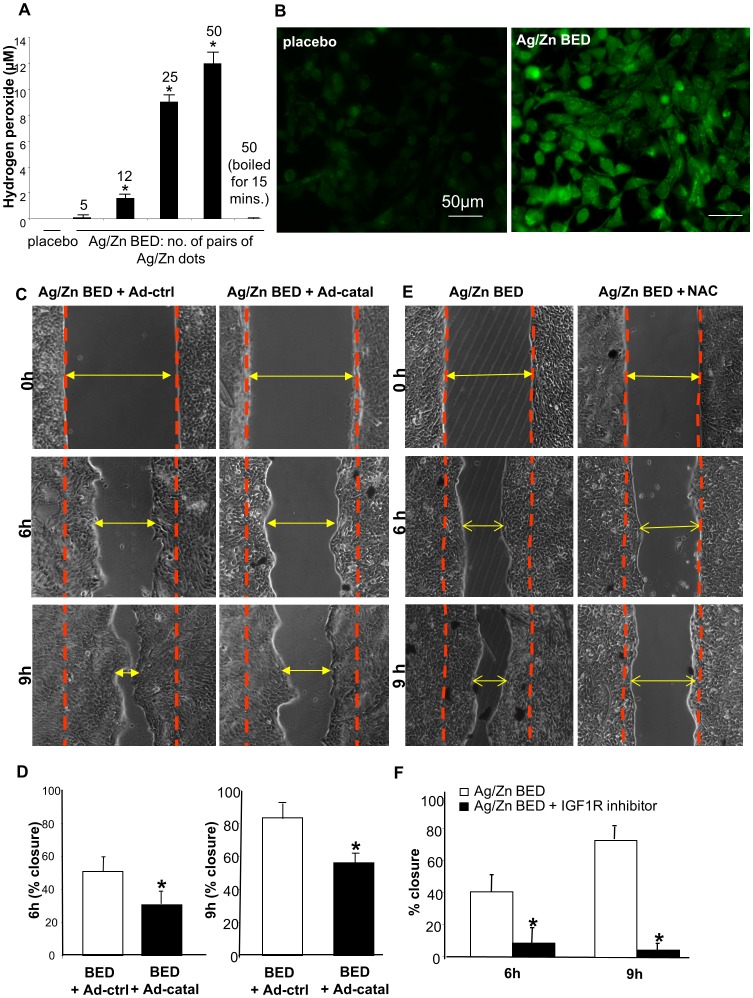
Increased generation of H_2_O_2_ under the effect of a Ag/Zn bioelectric dressing. (**A**) H_2_O_2_ production on immersing placebo/BED in PBS for 1 hr.H_2_O_2_ production increases with increase in the no. of Ag/Zn couples and attenuated on inactivating BED by boiling for 15 mins (n = 3). (**B**) HaCaT cells loaded by 5 µM PF6 (H_2_O_2_ indicator) showed increase in green fluorescence indicating generation of intracellular H_2_O_2_ (n = 3). (**C–D**) Ad-Catalase (Ad-Catal) attenuates Ag/Zn BED induced faster cell migration (n = 4). (**E–F**) N-acetyl cysteine attenuates Ag/Zn BED induced faster cell migration (n = 4).

Ag/Zn BED induces H_2_O_2_ production in PBS and the amount of H_2_O_2_ increases with the increase in the size of the dressing (number of Ag/Zn couples) and is not produced when the Ag/Zn BED is inactivated by boiling for 15 mins. (**Figure 4A** **and figure S1**). To study H_2_O_2_ production by cells, keratinocytes were cultured with BED or placebo for 24 h and then loaded with PF6-AM. PF6-AM (peroxyfluor-6 acetoxymethyl ester) is an indicator of endogenous H_2_O_2_ and detects changes in low levels of H_2_O_2_ in live cell settings and is nontoxic at the concentration used in the study [47], [48], [49], [50]. Significantly increased intracellular fluorescence was observed in keratinocytes exposed to BED compared to the placebo-treated cells. (**Figure 4B**). Over-expression of catalase, an enzyme that decomposes H_2_O_2_ blunted the effect of Ag/Zn BED on accelerated cell migration (**Figure 4C–D**). Treatment of keratinocytes with the antioxidant N-Acetyl Cysteine which also negated the effect of BED on accelerated cell migration (**Figure 4E–F**). Taken together, these data recognize H_2_O_2_ signaling as a key mediator of BED dependent acceleration of keratinocyte migration.

Wounds generate micromolar amounts of H_2_O_2_, primarily via NOx. Consistent, with evidence that low amperage DC currents can generate H_2_O_2_ by electrolysis [51] and H_2_O_2_ signaling is required for cell migration [52],[53], we observed that decomposition of H_2_O_2_ by catalase or antioxidant N-acetyl cysteine delivery, abolished the effect of Ag/Zn BED on cell migration. Therefore, BED may be used as an effective strategy to generate low levels of hydrogen peroxide over time.

### Ag/Zn BED improves mitochondrial function

**Figure 5:**
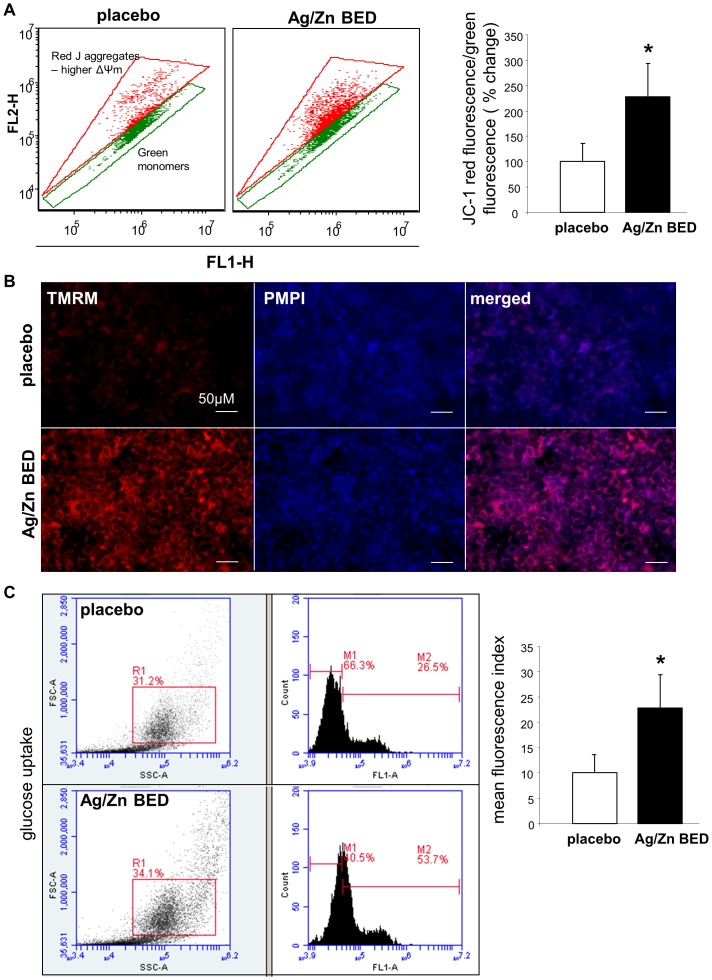
Ag/Zn bioelectric dressing energizes mitochondria in keratinocytes. (**A**) After 24 h of treatment with Ag/Zn BED, HaCaT cells were stained with JC-1 dye and analyzed using a flow cytometer (n = 3). (**B**) After 24 h of treatment with Ag/Zn BED, HaCaT cells were stained with TMRM and PMPI and fluorescence was observed using a Zeiss microscope. Increased relative mitochondrial membrane potential was observed under the Ag/Zn BED indicated by higher red fluorescence (n = 3).[TMRM = Tetramethylrhodamine methyl ester; PMPI = plasma membrane potential indicator] (**C**) Increased glucose uptake in Ag/Zn BED treated HaCaT cells (n = 3).

Cell migration is a process that demands higher energy supply [28], [54]. It is proposed that the superoxide radical can act as an electron donor for oxidative phosphorylation [55] and therefore may contribute to enhance mitochondrial membrane potential. External electrical stimulus may improve performance of the TCA cycle [56]. The hyper-activated TCA cycle is then expected to generate more NADH and FADH_2_ to enter into the electron transport chain and elevate the mitochondrial membrane potential (Δψm) [57]. Fluorescent dyes JC-1 and TMRM were used to measure mitochondrial membrane potential. JC-1 is a lipophilic dye which displays a red fluorescence with high Δψm and green fluorescence when Δψm is low, and TMRM which gives a red fluorescence proportional to Δψm, while PMPI measures plasma membrane potential [TMRM = Tetramethylrhodamine methyl ester; PMPI = plasma membrane potential indicator]. Treatment of keratinocytes with Ag/Zn BED for 24 h demonstrated significantly high red fluorescence with both JC-1 (**Figure 5A**) and TMRM (**Figure 5B**). These observations indicate that ES improved mitochondrial membrane potential. As a potential sequelae of a hyper-active TCA cycle, the pool of pyruvate which is the primary substrate for TCA cycle is depleted resulting in an enhanced rate of glycolysis in order to replenish the pyruvate pool [57]. Such enhanced rate of glycolysis may lead to an increase in glucose uptake in order to feed the glycolytic pathway forward. We therefore investigated the rate of glucose uptake in HaCaT cells treated with Ag/Zn BED. More than two fold enhancement of basal glucose uptake was observed after treatment with Ag/Zn BED for 24 h as compared to placebo treated control (**Figure 5C**).

### Ag/Zn BED induces IGF1-1 receptor phosphorylation, reduces cellular protein thiols and induces integrin_av_ expression

**Figure 6:**
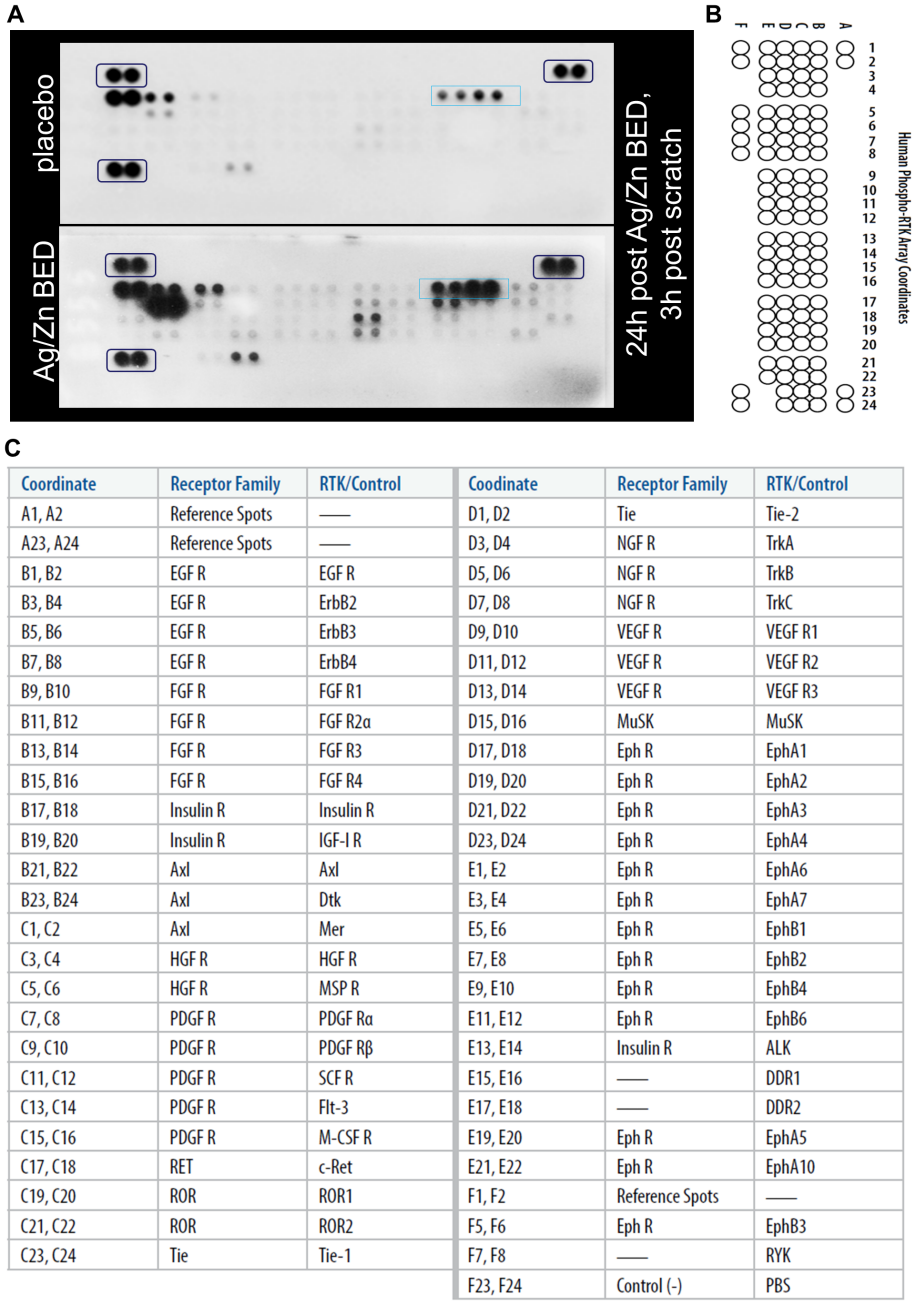
Increase in phosphorylation of IGF1 receptor under Ag/Zn bioelectric dressing. (**A**) Receptor tyrosine kinase phosphorylation array was performed on lysate from HaCaT cells treated with Ag/Zn BED for 24 h. Black boxes denote housekeeping controls, blue box denotes IGF1R. (**B–C**) Template for receptor tyrosine kinase phosphorylation assay.

**Figure 7:**
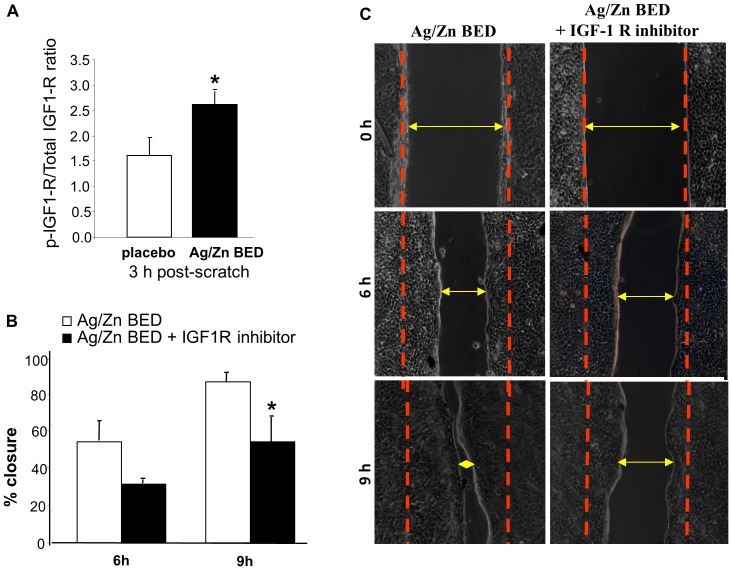
BED induces IGF1 receptor phosphorylation which helps in migration. (**A**) Validation of increased phosphorylation of IGF1R using ELISA. (**B–C**) IGF1R inhibitor attenuates Ag/Zn BED induced faster keratinocyte migration (n = 3).

Keratinocyte migration is known to involve phosphorylation of a number of receptor tyrosine kinases [58], [59], [60], [61], [62], [63]. To investigate which specific RTKs are activated as a result of Ag/Zn BED, scratch assay was performed on keratinocytes treated with Ag/Zn BED or placebo for 24 h. Samples were collected after 3 h and an antibody array that allowed simultaneous assessment of the phosphorylation status of 42 RTKs (human phospho-RTK array, R&D Systems) was used to quantify RTK phosphorylation. This approach led to the observation that Ag/Zn BED significantly induces IGF1R phosphorylation (**Figure 6**). This finding was verified by sandwich ELISA using an antibody against phospho-IGF1R and total IGF1R. As observed with the RTK array screening, potent induction in phosphorylation of IGF1R was observed 3 h post scratch under the influence of Ag/Zn BED (**Figure 7A**). Treatment with an IGF1R inhibitor blunted the accelerated keratinocyte migration observed in response to Ag/ZN BED treatment (**Figure 7B–C**).

**Figure 8:**
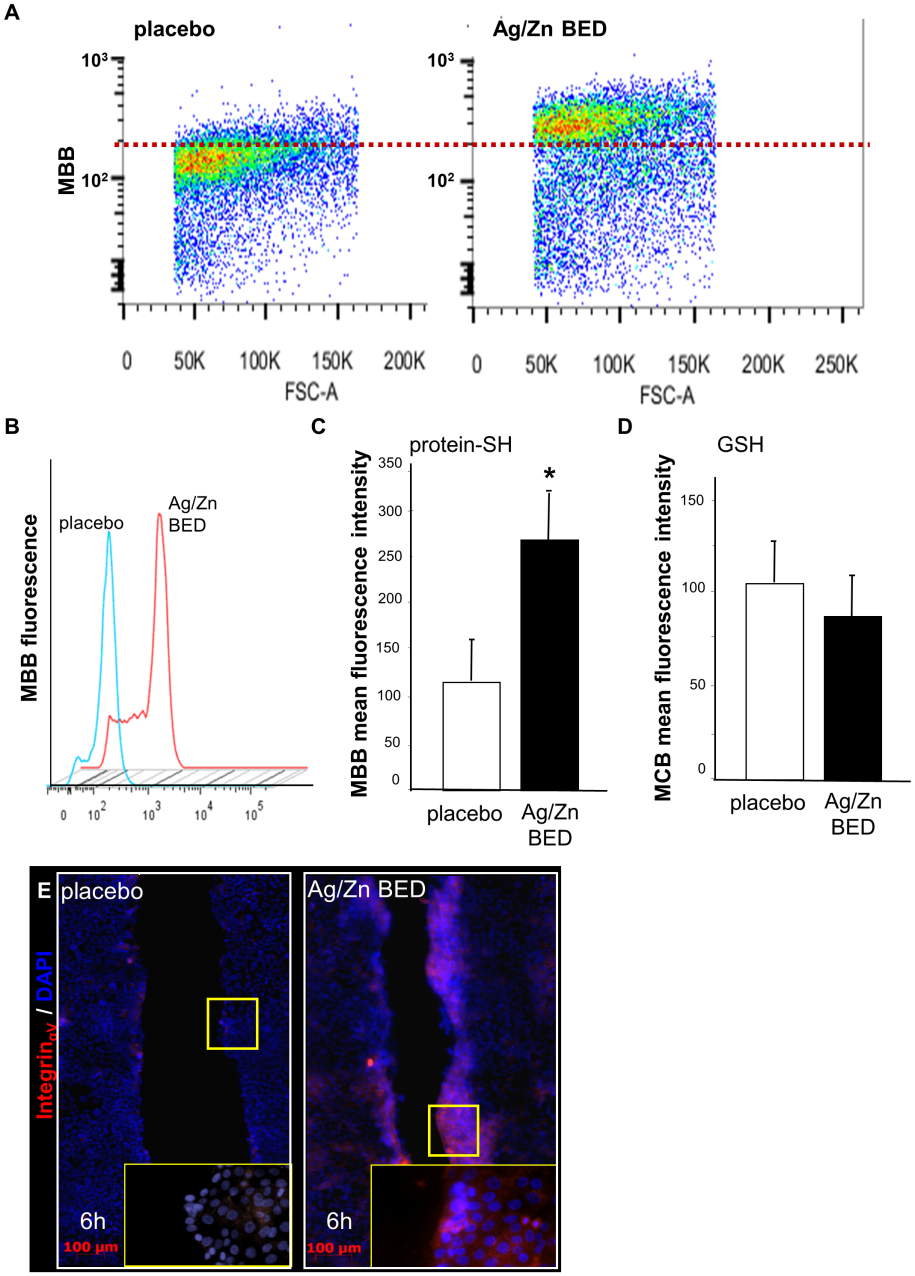
Ag/Zn BED increases integrin_αV_ expression and protein thiol in keratinocytes but does not increase glutathione. (**A–C**) Flow cytometry analysis demonstrates increase in fluorescence in HaCaT cells loaded with monobromobimane (MBB). (**D**) No significant change in fluorescence was observed in HaCaT cells loaded with monochlorobimane (MCB) (n = 4). (**E**) Integrin_αv_ expression at the scratch edge under placebo or Ag/Zn BED 6 h post scratch (n = 3).

MBB (monobromobimane) alkylates thiol groups, displacing the bromine and adding the fluorescent tag (λemission = 478 nm) to the thiol while MCB (monochlorobimane) reacts with only low molecular weight thiols like glutathione [64]. Fluorescence emission from UV laser excited keratinocytes loaded with either MBB or MCB was followed for 30 min. Mean fluorescence collected from 10,000 cells showed a significant rightward shift of MBB fluorescence emission from cells (**Figure 8A–C**). However, no significant change in MCB fluorescence was observed (**Figure 8D**) indicating net reduction of total protein thiols but not that of low molecular weight glutathione. HaCaT cells were treated with Ag/Zn BED for 24 h followed by a scratch and integrin expression was observed by immuno-cytochemistry at different time points. Significantly higher integrin expression was observed 6 h post scratch at the migrating edge of BED exposed keratinocytes (**Figure 8E**).

To recognize and to adhere to the extracellular matrix scaffold secreted by fibroblasts, keratinocytes depend on heterodimeric cell surface receptors of the family of integrins. Integrin receptors also allow cells to sense the mechanical condition of the extracellular environment, responding by intracellular signaling, triggering cell migration events. Integrin_αv_ directly associates with receptor tyrosine kinase IGF1R [65], modulates IGF1 receptor signaling [66] and drives keratinocyte locomotion [67], [68]. This work presents the first evidence demonstrating that ES induces integrin expression. Furthermore, it has been reported that integrins are rich in vicinal dithiols and are required for activation of integrins [69]. Thus, ES may serve as a potent driver of integrin function. We also note in our RTK array, that BED differentially regulated other receptor tyrosine kinases (**Figure 6**). A number of other pathways such as HGFR, VEGFR, EGFR, Rho/Rac was also observed to be differentially expressed under the effect of the BED. The effectiveness of these pathways in BED induced cell migration requires future studies.

## Conclusion

**Figure 9:**
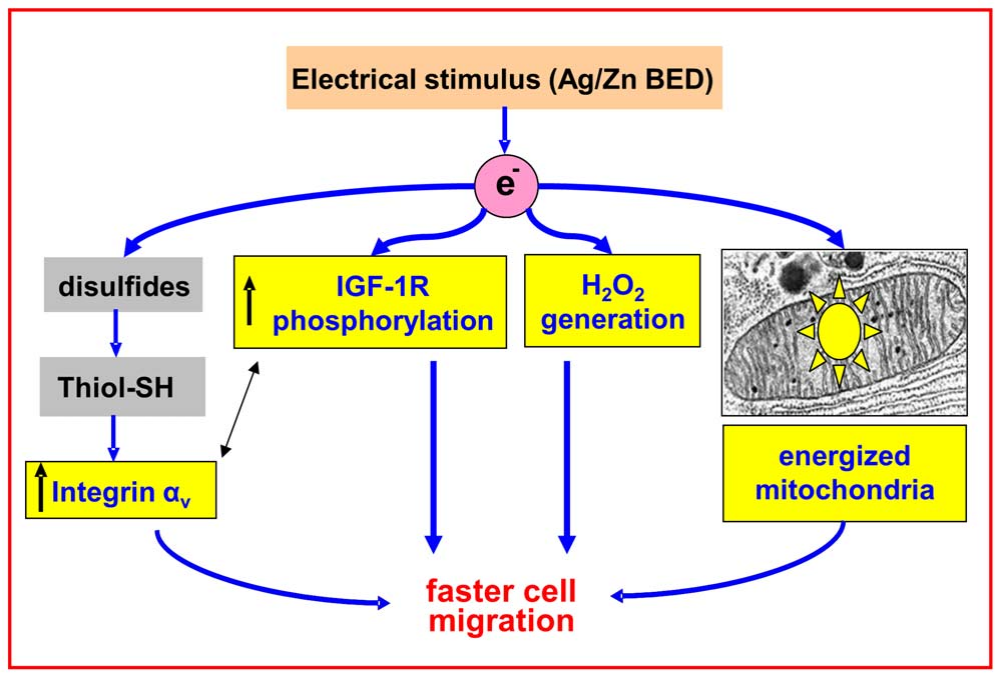
Summary figure.

In summary, we define in this work a Ag/Zn BED and establish that the electric field generated can cross-talk with keratinocytes via redox-dependent processes, thus improving keratinocyte migration (**Figure 9**). The importance of electrical control of cell physiology and the existence of injury potential was first demonstrated through the famous frog nerve-muscle experiments of Galvani in the mid-1700s [14]. Since then, application of bioelectricity in human healthcare has risen and has now been established in corneal repair, cancer metastasis, cell-cell communication, epithelial cell proliferation, axis of cell division, nerve growth and wound healing [14], [70]. In 2002, the Centers for Medicare and Medicaid Services approved reimbursement for use of ES in a clinical setting for certain chronic wounds that had failed standard wound therapies for diabetic, pressure ulcers (stage III or IV), stasis, and arterial ulcers. Guidelines on pressure ulcer treatment recommends ES for the management of recalcitrant stage II–IV pressure ulcers [71]. Several randomized controlled clinical trials have also demonstrated that ES combined with standard wound care improves the healing rate of chronic wounds more than standard wound care alone [6].

## Supporting Information

Figure S1 **Ag/Zn BED induces H_2_O_2_ production.** In PBS, Ag/Zn BED induces H_2_O_2_ production which increases with the increase in the size of the dressing (pairs of Ag/Zn dots) and is not produced when the Ag/Zn BED is inactivated by boiling for 15 mins.
(TIF)
