# Supplementary figures and images for: Improvement of Human Keratinocyte Migration by a Redox Active Bioelectric Dressing
Source: PLoS One. 2014 Mar 3;9(3):e89239. doi: 10.1371/journal.pone.0089239 (PMC3940438; doi:10.1371/journal.pone.0089239)

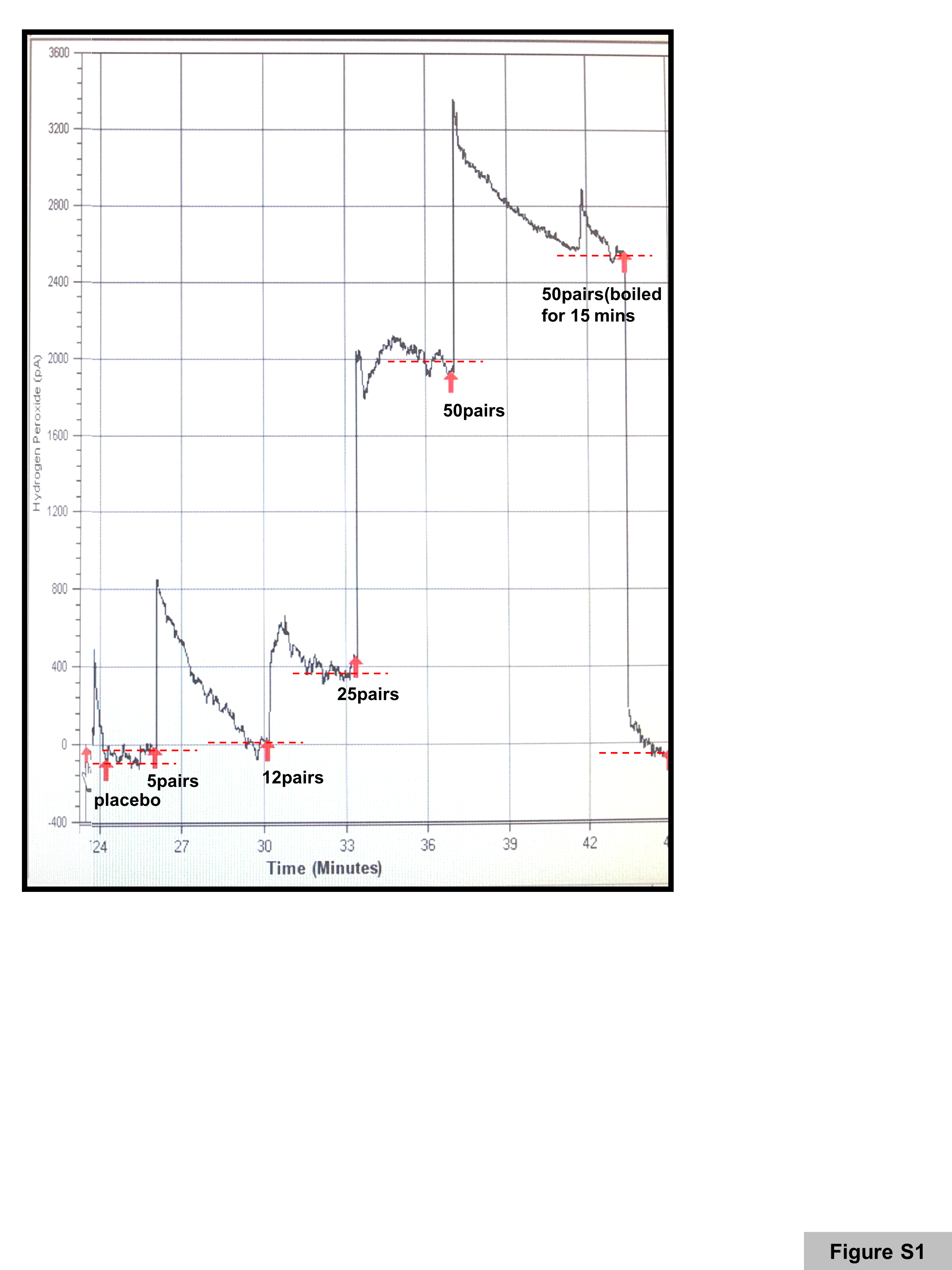

Supplement: Figure S1 — Ag/Zn BED induces H2O2 production. In PBS, Ag/Zn BED induces H2O2 production which increases with the increase in the size of the dressing (pairs of Ag/Zn dots) and is not produced when the Ag/Zn BED is inactivated by boiling for 15 mins. (TIF) [file pone.0089239.s001.tif]
